# Neuromodulation as an anticancer strategy

**DOI:** 10.1002/cam4.6624

**Published:** 2023-10-17

**Authors:** Ashwin Kumaria, Keyoumars Ashkan

**Affiliations:** ^1^ Department of Neurosurgery, Queen's Medical Centre Nottingham University Hospitals Nottingham UK; ^2^ Department of Neurosurgery King's College Hospital London UK

## Abstract

There may be a role for vagus nerve stimulation as an anticancer strategy. This may include the modulation of signalling between nerves and tumour cells and epigenetic influences, in addition to reduction of oxidative stress, anti‐inflammatory and endocrine mechanisms.

To the editor,

Despite advances in the development and clinical translation of anticancer strategies, cancer remains a major health concern. Neuromodulation is defined by the International Neuromodulation Society as: ‘the alteration of nerve activity through targeted delivery of a stimulus, such as electrical stimulation or chemical agents, to specific neurological sites in the body’.^1^ A role for neuromodulation as an anticancer strategy has previously been proposed in general,^2^ as well as specifically in the form of vagus nerve stimulation (VNS).^3^, ^4^

We read with great interest the excellent recent article of Abdullahi and colleagues.^5^ Here, they put forth a role for noninvasive VNS as a novel therapy in cancer and describe areas of cancer pathophysiology and immunology where vagal neuromodulation may be beneficial.^5^ We commend the authors on an impressive paper; the comments and observations that follow seek to provide alternative and additional perspectives to strengthen a case for neuromodulation as a novel treatment strategy in cancer.

VNS is a conventional treatment for medically refractory epilepsy and VNS‐mediated modulation of neuronal activity is thought to underlie this effect to a large extent.^6^ Subsequent to its demonstrated antiepileptic benefits, VNS has found newer indications in a multitude of neurological and psychiatric disorders by targeting excessive or abnormal neuronal activity.^7^, ^8^ Such abnormal neuronal activity has been found in brain tumours—both primary malignant gliomas^9^, ^10^ and brain metastases^11^—and we have previously described a role for neuromodulation, including VNS as a novel therapeutic strategy in brain tumours.^4^ However, emerging evidence suggests that neuronal activity may contribute to the pathophysiology of other cancers by promoting their growth.^12^, ^13^ Attenuating the neuronal activity that drives cancer growth may be another mechanism by which VNS could exert an anticancer effect.

Furthermore, some cancers are neurotropic and spread by perineural invasion, for example pancreatic, prostatic, colorectal, breast, head and neck cancers.^14^, ^15^ It is probable that paracrine signalling between neural tissue and tumour cells may contribute to the phenomenon of perineural invasion and their propagation in general.^14^ Targeting perineural tumour invasion may represent a further therapeutic opportunity in VNS.

Additionally, vagal stimulation has been shown to induce widespread systemic epigenetic effects which may have an antitumour influence.^16^ Alterations in DNA methylation and histone modification have been characterised in stress‐response signalling cascades as well as in multiple inflammatory and homeostatic pathways.^16^ These epigenetic changes may represent a parallel effector of the established anti‐inflammatory effect associated with VNS through activation of the cholinergic anti‐inflammatory pathway.^17^ Epigenetic influences as such may contribute to a potential anticancer effect of VNS.

Taken together, there may be a role for VNS as an anticancer strategy. This may include the modulation of signalling between nerves and tumour cells and epigenetic influences, in addition to reduction of oxidative stress, anti‐inflammatory and endocrine mechanisms. Other neuromodulation modalities may also provide similar anticancer inputs, including deep brain stimulation, spinal cord stimulation, peripheral/cranial nerve stimulation, transcranial magnetic stimulation, pharmacotherapies and others. The wider field of ‘cancer neuroscience’ is being actively researched.^18^ A potential development would be that ‘neuromodulation’ becomes a class of anticancer treatments of the future alongside conventional treatments such as surgery, chemotherapy, radiotherapy and immunotherapy.

## AUTHOR CONTRIBUTIONS

**Ashwin Kumaria:** Conceptualization (lead); data curation (lead); formal analysis (lead); investigation (lead); methodology (lead); project administration (equal); software (lead); supervision (equal); validation (lead); visualization (lead); writing – original draft (lead); writing – review and editing (equal). **Keyoumars Ashkan:** Project administration (equal); supervision (equal); writing – review and editing (equal).

## Data Availability

Data sharing is not applicable to this article as no new data were created or analyzed in this study.
